# *Phyllanthus emblica* (Amla) Fruit Powder as a Supplement to Improve Preweaning Dairy Calves’ Health: Effect on Antioxidant Capacity, Immune Response, and Gut Bacterial Diversity

**DOI:** 10.3390/biology11121753

**Published:** 2022-12-01

**Authors:** Mebrahtom Nguse, Yi Yang, Zilin Fu, Jianchu Xu, Lu Ma, Dengpan Bu

**Affiliations:** 1 State Key Laboratory of Animal Nutrition, Institute of Animal Science (IAS), Chinese Academy of Agricultural Sciences (CAAS), Beijing 100193, China; 2 Department of Animal Sciences (ARWS), College of Dryland Agriculture and Natural Resources, Mekelle University, Mekelle P.O. Box 231, Ethiopia; 3 World Agroforestry Center, East and Central Asia, Kunming 650201, China; 4 Joint Laboratory on Integrated Crop-Tree-Livestock Systems of the Chinese Academy of Agricultural Sciences (CAAS), Ethiopian Institute of Agricultural Research (EIAR) and World Agroforestry Center (ICRAF), Beijing 100193, China

**Keywords:** *Phyllanthus emblica*, Amla, tannins, oxidative stress, antioxidant, immune response

## Abstract

**Simple Summary:**

Disease is among the leading problems in calf rearing, and Amla fruit powder could be a good candidate to improve calf immunity and resistance against infections due to its antioxidant, antimicrobial, and immunomodulatory effects. The aim of this study was to evaluate the effect of Amla fruit powder supplementation on antioxidant capacity, immune response, and gut microbial diversity of preweaning dairy calves. Supplementation of Amla fruit powder at 5 g per day improved the antioxidant capacity and immune response of preweaning dairy calves, while higher doses (20 and 40 g per day) negatively affected the antioxidant capacity and immune response. Ten grams per day supplementation showed comparable results to the control. Thus, 5 g per day Amla supplementation could be recommended for preweaning dairy calves.

**Abstract:**

Disease is the main reason for the use of antimicrobials in calf rearing, and antibiotics are commonly used to treat calves, including for unknown diseases. This leads to antimicrobial resistance, which is a challenge to the livestock industry and public health. Plant products containing high levels of phytochemicals may improve the immunity and resistance of calves against infections, thereby reducing the use of antimicrobials. This study aimed to investigate the effect of *Phyllanthus emblica* (Amla) fruit powder (PE) supplementation on antioxidant capacity and immune response of preweaning dairy calves. One hundred, 2-day-old, male Holstein calves were randomly assigned into five treatment groups receiving 0, 5, 10, 20, and 40 g/d PE supplementation. Antioxidant and immune indices and pro- and anti-inflammatory cytokines were analyzed from serum samples, whereas 16S rRNA was analyzed from rumen fluid and fecal samples. PE supplementation, at 5 g/d, protected calves against oxidative stress and improved antioxidant enzymes and immune and anti-inflammatory responses, showing its immunity-enhancing and protective roles against infections. However, the antioxidant capacity and immune response decreased with increasing PE levels, illustrating the adverse effects of PE supplementation at higher doses. The analysis of ruminal and fecal bacterial community abundance detected higher proportions of *Firmicutes* at an early age, and a higher *Bacteroidetes* to *Firmicutes* ratio at weaning, in calves supplemented with 5 g/d PE. This contributed to the development of the immune system in early life, and improved immune and anti-inflammatory responses at a later age. The overall results suggest that PE could be supplemented at 5 g/d for preweaning dairy calves to protect against oxidative stress and infections while maintaining normal gut microbial hemostasis.

## 1. Introduction

Disease is among the leading causes of calf diarrhea [1,2], which in turn leads to the use of antimicrobials in calf rearing [3]. Antibiotics are the most commonly used antimicrobial in preweaning dairy calves [4], including to treat unknown diseases [5]. Improper or misuse of antimicrobials leads to antimicrobial resistance [6], a challenge to the livestock industry and public health. As a result of the global concern over antimicrobial resistance, alternative solutions, such as plant origin additives (phytochemicals), have therefore drawn the attention of many researchers in the last one and a half decades [7], and are gaining roles in the livestock industry to enhance the health condition of animals [8]. Although total replacement of antibiotics is not likely to happen in the near future [6], phytochemicals may play preventive (phytochemoprophylactic) and therapeutic (phytochemotherapeutic) roles [7] to enhance the health status of animals, and thereby reduce the use of antimicrobials.

Amla (*Phyllanthus emblica*) fruit is among the natural sources of antioxidants and nutraceuticals of medicinal value due to its high contents of phytochemical compounds [9]. It is a small- to medium-sized deciduous plant known for its essential role in traditional ayurvedic medicine [10], with the fruit being the most useful part in this regard [11]. Amla fruit is rich in phytochemical compounds such as phenolics, flavonoids, tannins, saponins, alkaloids, carbohydrates, and organic acids [12,13]. There are variations in the phytochemical contents of Amla fruit extracts reported in different studies, which could be due to the variety, source of the fruit, stage of development during harvest, and methods of collection, processing, and extraction. For instance, Sheoran et al. [14] reported about 240 mg/g gallic acid equivalents and 356 mg/g rutin equivalents of phenolic and total flavonoid contents, respectively, from Amla fruit extracts, whereas Saha and Verma [15] found about 21 mg gallic acid equivalents, 16 mg quercetin equivalent, and 12 mg rutin equivalents of phenolic, flavonoid, and tannin contents, respectively, per g dry weight of Amla fruit. We previously reported that phenolic acids, flavonoids, and tannins accounted for about half of the total phytochemical compounds in Amla whole-fruit powder [16].

Extracts from Amla fruit showed strong antioxidant [12,14], antimicrobial [14], anti-inflammatory [17,18], and immunomodulatory [19,20] properties; protected against intracellular oxidative stress in mammary cells [12]; and improved the survivability of buffalo calves [21]. These studies’ results suggest that Amla fruit could be a potential additive to improve the nutrition and health status of ruminants. However, there is lack of information on the effect of Amla fruit powder supplementation on the antioxidant capacity and immune response of dairy calves. It was, therefore, in line with these facts that we hypothesized that the supplementation of Amla (*Phyllanthus emblica*) fruit powder may improve the health status of preweaning dairy calves by enhancing the antioxidant capacity and immune responses. The objective of the study was to evaluate the effect of Amla (*Phyllanthus emblica*) whole-fruit powder supplementation on antioxidant capacity, immune response, and ruminal and fecal bacterial structure of preweaning dairy calves.

## 2. Materials and Methods

### 2.1. Diet Preparation, Experimental Animals, and Treatments

Preparation of Amla (*Phyllanthus emblica*) fruit powder (PE), experimental animals and housing, treatment diets, and feeding regimen were as reported in our previous paper [16]. Briefly, Amla fresh whole fruits, including the seeds, were sundried after being manually crushed by hammering. Sundried fruits were first crushed with a 6 mm sieve size cutting mill (Type SM100, Retsch GmbH, Haan, Germany), and then passed through a 1 mm sieve size ultracentrifugal mill (Type ZM200, Retsch GmbH) to produce the fruit powder.

One hundred, 2-day (d)-old, Holstein male calves (42.6 ± 1.7 kg body weight (BW)) were randomly assigned to one of the five treatments (n = 20 calves per treatment) using random numbers generated in Excel (Microsoft, Redmond, WA, USA). The treatments were control (CON), PE5, PE10, PE20, and PE40, with 0, 5, 10, 20, and 40 g/d/head supplementation of PE, respectively. Calves were selected based on the following preset criteria: (1) birth weight (41 ± 2 kg); (2) health and physical check by a veterinarian (only calves with no physical or health problems); and (3) birth record (only calves with no birth-related problems). Calves were housed in individual Calf-Tel hutches (2.2 m × 1.2 m × 1.3 m; Hampel Corp., Germantown, WI, USA) bedded with sand. The experiment was carried out from 2 days of age up to weaning at 75 days of age.

All calves received a total of 6 L of colostrum each at 1, 6, and 18 h after birth, 2 L each time, and were fed fresh whole milk from 2 to 7 days of age; milk replacer from 8 to 75 days of age; and pelleted calf starter (Table 1) from 2 to 75 days of age. PE was supplemented for the whole experiment period (2–75 days of age) by mixing into liquid feed twice a day, and was manually stirred to ensure it dissolved well. Esophageal feeders (for colostrum), plastic calf feeding bottles with a nipple (fresh milk for calves between 2 and 3 days of age), and individual buckets (fresh milk and/or milk replacer after 3 days of age, and calf starter) were used for feeding calves. PE supplementations were given mixed into liquid feed (stirred manually to dissolve it well) and in two equal amounts in the two meals (0700 and 1500 h). Mixing into the liquid feed was the preferred way for supplementation of PE because it allowed full dissolving and total uptake of the PE by the calves at each supplementation time.

Samples of calf starter, PE, and milk replacer were collected at weekly intervals and stored at −20 °C until the feed analysis. The samples were oven dried at 55 °C for 72 h before the analysis. Analyses of the chemical composition of feed samples were conducted in accordance with the AOAC [22] international methods, as follows: dry matter, method 930.15; crude protein, Kjeldahl (method 984.13); ash, method 942.05. Ether extract was analyzed according to Sun et al. [23], whereas acid detergent fiber and neutral detergent fiber contents were analyzed as described by Van Soest et al. [24]. Weekly samples of fresh whole milk were preserved with potassium dichromate and stored at −20 °C until the subsequent analysis of contents using a MilkoScan machine (Type 78,110; Foss Electric).

### 2.2. Blood Sample Collection and Analysis

About 10 mL of blood samples were collected using serum separator tubes from the jugular vein 2 h after morning feeding at 28 and 75 days of age. Blood samples were allowed to clot overnight at 4 °C temperature, and then centrifuged for 15 min at 3000× *g* and 4 °C temperature. The serum was transferred to 2 mL tubes and stored at −20 °C until subsequent analysis. Serum samples from all (100) calves were used for the analysis of the immune response and antioxidant capacity indices during each sampling time. For the analysis of immune indices, serum samples from two calves (in the same treatment) were mixed into one in equal proportion, and analyzed as one sample due to a limited number of ELISA kits.

Serum concentrations of malondialdehyde (MDA) and activities of glutathione peroxidase (GSH-Px), superoxide dismutase (SOD), and catalase (CAT) were analyzed by colorimetric methods using a spectrophotometer (microplate reader) according to the instructions of the respective commercial kits (Jiancheng Bioengineering Institute, Nanjing, China). Serum concentrations of immunoglobulin A (IgA), immunoglobulin G (IgG), immunoglobulin M (IgM), tumor necrosis factor α (TNF-α), and interleukin 10 (IL-10) were analyzed using Cusabio Biotech Co., Ltd. (Wuhan, China), Enzyme-Linked Immunosorbent Assay (ELISA) kits (Catalog number: CSB-E12015B, CSB-E12018B, CSB-E12017B, CSB-E12020B, and CSB-E12917B, respectively) according to the manufacturer’s instructions (www.cusabio.com, accessed on 31 March 2022). Briefly, microtiter plates, pre-coated with respective antibodies, were used, and a competitive inhibition enzyme immunoassay technique was employed. Commonly used, easy to use, precision, and timely availability were among the criteria employed to select the commercial kits used for the analysis of antioxidant and immune response indices in the study.

### 2.3. Rumen Fluid and Fecal Sample Collection

Rumen fluid samples were collected at 14, 42, and 75 days of age from 35 randomly selected calves (n = 7 per treatment) 2 h after morning feeding of liquid feeds, using a flexible stomach tube and pump (Anscitech Co. Ltd., Wuhan, China). To avoid contamination with saliva, the first 10 mL of the rumen fluid was discarded. Samples were squeezed through four layers of cheesecloth, and aliquots of the samples were placed in 2 mL tubes. Then, the samples were immediately stored in liquid nitrogen, transported to the laboratory, and frozen at −80 °C until subsequent analysis. Samples were collected from the same calves in all three sampling periods. Fecal samples were collected directly from the rectum by hand using long arm gloves at 2, 28, 42, and 75 days of age from the same calves as provided the rumen fluid samples (35 randomly selected calves (n = 7 per treatment)). One glove was used for each calf to avoid contamination. Feces samples were placed in 2 mL tubes and immediately stored in liquid nitrogen, then transported to the laboratory, and frozen at −80 °C until the analysis.

### 2.4. DNA Extraction, PCR Amplification, and 16S rRNA Sequencing

The microbial community of rumen fluid and fecal samples were analyzed at Shanghai Meiji Biomedical Technology Co., Ltd. (Shanghai, China) (www.majorbio.com, accessed from 30 September to 9 December, 2021, according to respective standard protocols. Briefly, microbial genomic DNA from rumen fluid and fecal samples were extracted using an E.Z.N.A.^®^ soil DNA Kit (Omega Bio-tek, Norcross, GA, USA) following the manufacturer’s instructions. NanoDrop 2000 UV-vis spectrophotometer (Thermo Scientific, Wilmington, NC, USA) was used to determine the concentration and purity of the DNA extract, and its integrity was checked on 1% agarose gel electrophoresis. The forward and reverse primers (338F, 5′-ACTCCTACGGGAGGCAGCAG-3′ and 806R, 5′-GGACTACHVGGGTWTCTAAT-3′, respectively) were used to amplify the V3–V4 hypervariable region of the bacterial 16S rRNA gene with an ABI GeneAmp^®^ 9700 PCR thermocycler (ABI, Los Angeles, CA, USA). The PCR products were separated on 2% agarose gel via electrophoresis, and purified and quantified using the AxyPrep DNA Gel Extraction Kit (Axygen Biosciences, Union City, CA, USA) and Quantus™ Fluorometer (Promega, Madison, WI, USA), respectively. Purified amplicons were pooled in equal concentrations for paired-end library construction, and paired-end sequencing was carried out on an Illumina MiSeq PE300 platform/NovaSeq PE250 platform (Illumina, San Diego, CA, USA) at Majorbio Bio-Pharm Technology Co. Ltd. (Shanghai, China) in accordance with the established protocols.

### 2.5. Sequencing Data Processing and Analysis

The online Majorbio Cloud Platform (www.majorbio.com, accessed on 11 April 2022), was used to analyze the bioinformatics data on ruminal and fecal microbiota [25]. The raw Illumina MiSeq sequencing reads were demultiplexed, quality controlled, and filtered by fastp version 0.20.0 [26], and FLASH version 1.2.11 [27] was used to merge the reads. In brief, the 300 bp reads were trimmed at any site receiving an average quality score of <20 over a 50 bp sliding window, and truncated reads shorter than 50 bp or containing ambiguous characters were discarded. The overlapped sequences of only those overlapping sequences longer than 10 bp were assembled. The overlap region’s maximum mismatch ratio was 0.2. Unassembled reads were removed; samples were separated based on the barcode and primers, and the sequence direction was modified (perfect barcode matching, two-nucleotide primer mismatch).

UPARSE version 11 [28] was used to cluster operational taxonomic units (OTUs) with 97% similarity cutoff [28,29], and chimeric sequences were found and eliminated. By applying a confidence level of 0.7, the RDP Classifier version 2.13 [30] was used to analyze each OTU representative sequence’s taxonomy against the 16S rRNA database (SILVA version 138).

### 2.6. Statistical Analysis

Before the statistical analyses, data were checked for normality and outliers using the PROC UNIVARIATE procedure of SAS (version 9.4, SAS Institute Inc., Cary, NC, USA). Statistical analyses of antioxidant and immune response variables were performed using the PROC MIXED procedure of SAS with the following model:

Yijk = µ + Ai + Tj + Dk + (Tj × Dk) + εijk,(1)

where Yijk is the response variable (all antioxidant and immune response variables), µ is overall mean, Ai is random effect of calf, Tj is treatment (different levels of PE and CON groups), Dk is day (age in weeks), Tj × Dk is treatment–day interaction, and εijk is random error. Calf and day were considered as random and repeated effects, respectively; the fixed effects were treatment, day, and treatment × day interaction. Pretest results of the current study were used to calculate the statistical power using the PROC POWER procedure of SAS. Using means, standard deviations, α = 0.05, and 20 calves per treatment, we found the statistical power to be more than 90% for the antioxidant capacity indices. Statistical significance was declared at *p* ≤ 0.05 and tendencies at 0.05 < *p* ≤ 0.10.

## 3. Results

### 3.1. Antioxidant Capacity

**Figure 1:**
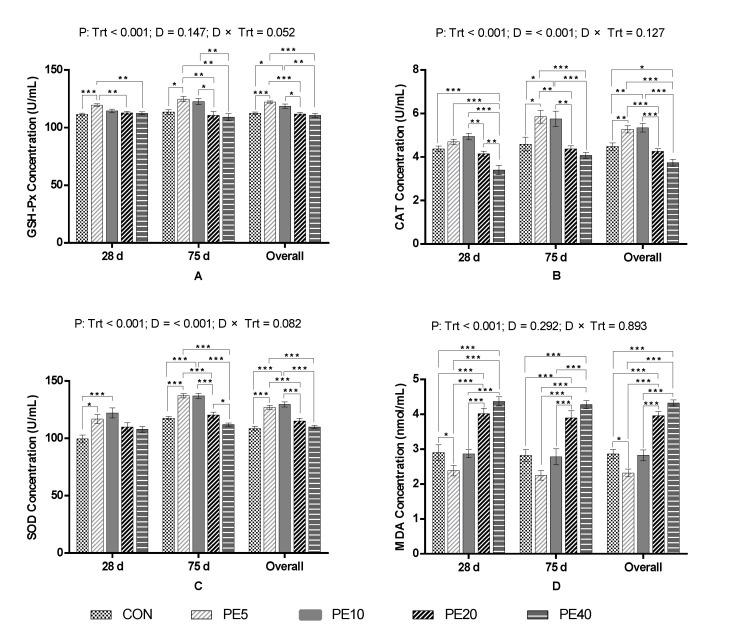
Serum antioxidant activities of calves supplemented with Amla (*Phyllanthus emblica*) whole-fruit powder: (**A**) glutathione peroxidase (GSH-Px), (**B**) catalase (CAT), (**C**) superoxide dismutase (SOD), (**D**) malondialdehyde (MDA). CON, PE5, PE10, PE20, and PE40 indicate control (0), 5, 10, 20, and 40 g/d/head supplementation of Amla fruit powder, respectively. Trt = treatment; D = day; Trt × D = treatment–day interaction. The values are mean ± SEM, and *p*-values shown are for the “overall” result; significant differences are marked as * 0.01 < *p* ≤ 0.05, ** 0.001 < *p* ≤ 0.01, *** *p* ≤ 0.001.

The current study reveals that supplementation of 5 g/d Amla (*Phyllanthus emblica*) fruit powder (PE5) significantly improved the activities of GSH-Px (*p* < 0.001) and SOD (*p* = 0.011) and reduced serum concentration of MDA (*p* = 0.037), whereas the activity of CAT was not affected (*p* > 0.05), compared to the CON, in dairy calves at 28 days of age (Figure 1). At the same time, GSH-Px, CAT, and MDA in PE10 were comparable to the CON (*p* > 0.05), while SOD was significantly higher (*p* < 0.001). Similarly, PE20 and PE40 did not affect the activities of GSH-Px and SOD (*p* > 0.05), but significantly increased the serum concentration of MDA (*p* < 0.05), compared to the CON. Activity of CAT was significantly lower in PE40 than in all groups (*p* < 0.05).

At day 75, serum from calves supplemented with PE5 had higher activities of GSH-Px (*p* = 0.035), CAT (*p* = 0.012), and SOD (*p* < 0.001) compared to the CON group, whereas MDA was not significant (*p* > 0.05). PE10 had comparable activity of GSH-Px and concentration of MDA (*p* > 0.05), but significantly higher activities of CAT (*p* = 0.026) and SOD (*p* < 0.001), compared to the CON. The activities of GSH-Px, CAT, and SOD in PE20 and PE40 were similar to the CON (*p* > 0.05), but the concentration of MDA was significantly higher (*p* < 0.05). The overall study period antioxidant capacity of preweaning calves supplemented with PE illustrates that PE5 significantly improved the activities of GSH-Px (*p* < 0.001), CAT (*p* = 0.008), and SOD (*p* < 0.001), and reduced the concentration of MDA (*p* = 0.011) in serum, compared to the CON. Similarly, PE10 improved the activities of GSH-Px (*p* = 0.037), CAT (*p* = 0.003), and SOD (*p* < 0.001), but not the concentration of MDA (*p* > 0.05). However, higher doses (PE20 and PE40) did not affect the activities of GSH-Px or SOD, but increased the concentration of MDA (*p* < 0.05) compared to the CON. PE40 also reduced the activity of CAT (*p* = 0.015) compared to the CON.

### 3.2. Immune and Anti-Inflammatory Responses

The immune responses of preweaning calves supplemented with PE are shown in Table 2. At 28 days of age, PE5 improved the serum concentration of IgG (*p* = 0.042), IgM (*p* = 0.039), and IL-10 (*p* = 0.028), but IgA and TNF-α were not affected (*p* > 0.05), compared to the CON. PE10, PE20, and PE40 did not affect IgA, IgG, IgM, TNF-α, or IL-10 compared to the CON (*p* > 0.05), with the exception that PE10 tended to have higher IgG (*p* = 0.064) and IgM (*p* = 0.054) than the CON. However, concentrations of IgA and IgG for PE10 were higher than for PE20 and PE40 (*p* < 0.05). IgM was also higher in the PE10 group than PE40 (*p* = 0.034). At day 75, PE5 improved the concentrations of IgA (*p* = 0.032), IgG (*p* = 0.031), IgM (*p* = 0.038), and IL-10 (*p* = 0.038), but not TNF-α (*p* = 0.081), whereas PE10, PE20, and PE40 did not affect any of the parameters (*p* > 0.05) compared to the CON. Comparison among the different levels of PE supplementation revealed that PE20 and PE40 groups had significantly lower serum concentrations of IgA and IgM and higher TNF-α than in PE5 and PE10 (*p* < 0.05). The concentrations of IgG in PE20 and IL-10 in PE40 were also lower than in PE5 and PE10 (*p* < 0.05).

The overall study period mixed model analysis results show that PE5 improved the serum concentrations of IgA (*p* = 0.002), IgG (*p* < 0.001), IgM (*p* = 0.001), and IL-10 (*p* < 0.001), but did not affect TNF-α (*p* > 0.05) in preweaning dairy calves, compared to the CON. Likewise, PE10 improved IgA (*p* = 0.011), IgG (*p* = 0.006), IgM (*p* = 0.010), but did not affect TNF-α (*p* > 0.05) or IL-10 (*p* = 0.0515), compared to the CON. The overall concentrations of IgM and IL-10 in PE20 and PE40 were comparable to the CON (*p* > 0.05). However, the IgA in PE40 and IgG in PE20 and PE40 were significantly lower than in the CON (*p* < 0.05), while TNF-α was higher in PE40 (*p* = 0.049).

### 3.3. Ruminal and Fecal Bacterial Structure

#### 3.3.1. Diversity and Richness of Bacterial Flora

After the two-terminal sequence quality control stitching, 12,695,768 sequences with an average sequence length of 415 bp were generated from 245 rumen fluid and fecal samples. A total of 1217 species of bacteria belonging to 621 genera and 228 families were identified and classified into 2956 OTU based on 97% similarity. *Firmicutes*, *Bacteroidota*, *Proteobacteria*, and *Actinobacteriota* were the dominant phyla, while *Escherichia-Shigella*, *Prevotella*, *norank_f__norank_o__Clostridia_UCG-014*, *Collinsella*, and *Bacteroides* were the top five at the genus level. The Simpson and Shannon indices were used to compare the bacterial diversity, while the Sobs and abundance-based coverage estimator (ACE) indices were used to compare the richness among treatment groups.

**Figure 2:**
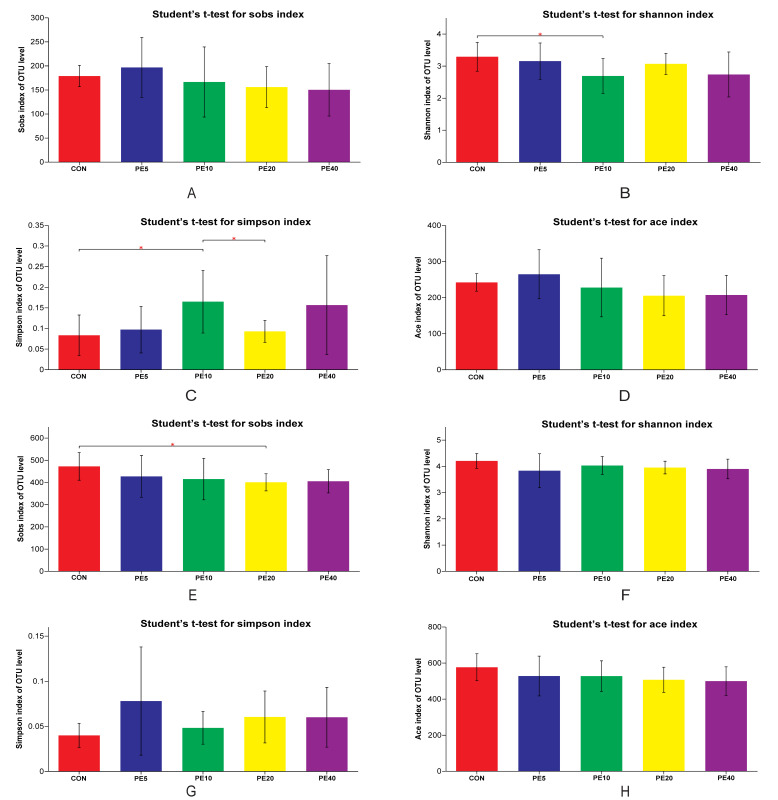
Fecal bacterial diversity of calves supplemented with Amla (*Phyllanthus emblica*) whole-fruit powder at 28 (**A**–**D**) and 75 (**E**–**H**) days of age. CON, PE5, PE10, PE20, and PE40 indicate control (0), 5, 10, 20, and 40 g/d/head supplementation of Amla fruit powder, respectively. Significant differences are marked as * 0.01 < *p* ≤ 0.05.

The alpha diversity analysis of the fecal bacterial community at 28 days of age (Figure 2A–D) revealed that PE10 had higher diversity (Simpson index) than the CON (*p* = 0.035) and PE20 (*p* = 0.036), whereas the Shannon index was higher in CON than PE10 (*p* = 0.044). There was no difference among other treatment groups (*p* > 0.05). Moreover, the bacterial richness ACE index at 28 d did not show any difference (*p* > 0.05) among all treatment groups. The Simpson and ACE indices of the fecal bacterial community at 2 and 42 days of age were similar (*p* > 0.05) among all groups (Supplementary Figure S1). At weaning (75 d), the CON group had higher bacterial richness (Sobs index) than PE20 (*p* = 0.024), while the ACE index (*p* = 0.098) tended to decrease in the PE20 group compared to the CON (Figure 2E,H). However, the Shannon and Simpson indices did not show any significant differences (*p* > 0.05) among all groups (Figure 2F,G). The overall mixed model analysis further showed that treatment did not affect the Simpson, Shannon, ACE, or Sobs indices, and no treatment–day interaction effect was detected (*p* > 0.05). However, day significantly affected the Simpson, Shannon, ACE, and Sobs indices; 2 d had a higher Simpson index but lower Shannon, ACE, and Sobs indices than 28, 42, and 75 d (*p* < 0.05).

No significant difference was detected (*p* > 0.05) for the rumen bacterial diversity or richness at 14, 42, and 75 days of age (Supplementary Figure S2). There was only an increasing trend in the ACE index at 42 d, and a decreasing trend in the Simpson index at 75 d, with increasing level of PE supplementation.

#### 3.3.2. Difference in the Abundance of Bacterial Taxa among the Treatments

**Figure 3:**
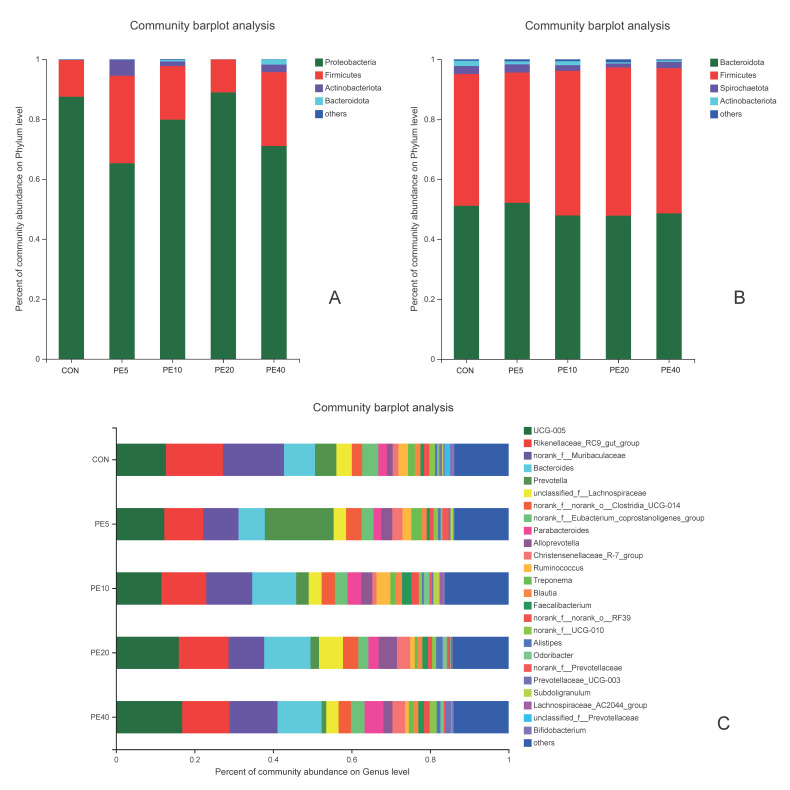
Relative abundance of fecal bacterial community of calves supplemented with Amla (*Phyllanthus emblica*) whole-fruit powder at (**A**) 2 days of age on phylum level; (**B**) 75 days of age on phylum level; (**C**) 75 days of age on genus level. CON, PE5, PE10, PE20, and PE40 indicate control (0), 5, 10, 20, and 40 g/d/head supplementation of Amla fruit powder, respectively.

The community abundance at the phylum level at early age (2 d) showed that *Proteobacteria* dominated the fecal bacterial community, followed by *Firmicutes* and small proportions of *Actinobacteriota* and *Bacteroidota* in all treatments (Figure 3A). The proportion of *Proteobacteria* in PE5 was 22% lower than in CON, whereas the proportion of *Firmicutes* was 16.9% higher in PE5 than in CON. At 28 and 42 d, the fecal bacterial community abundance shifted towards a higher proportion of *Firmicutes,* followed by *Actinobacteriota* and *Bacteroidota*, in all treatments (Supplementary Figure S3A,B). The proportion of *Firmicutes* at 42 d was 10.2%, 24.3%, 18.1%, and 21.2% higher in PE5, PE10, PE20, and PE40, than the CON. At weaning (75 d), *Bacteroidota* and *Firmicutes* were the dominant phyla. The fecal *Bacteroidota* to *Firmicutes* ratios were 1.10, 1.20, 0.99, 0.97, and 1.00 in the CON, PE5, PE10, PE20, and PE40 groups, respectively (Figure 3B). *UCG-005*, *Rikenellaceae_RC9_gut_group*, *norank_f__Muribaculaceae*, *Bacteroides*, and *Prevotella* were the dominant fecal bacterial genera at 75 d (Figure 3C). At this time, the genus *Prevotella* tended to be higher (*p* = 0.098) in PE5 (17.5% proportion) compared to the other groups, which accounted 5.4, 3.2, 2.2, and 1.2% in CON, PE10, PE20, and PE40 groups, respectively.

The ruminal bacterial community at 14 d at the phylum level was dominated by *Bacteroidota* and *Firmicutes,* followed by *Actinobacteriota*, *Proteobacteria*, and *Desulfobacterota*, in all treatments (Supplementary Figure S4A). At 75 d, *Firmicutes* was the dominant phylum in all treatment groups (Supplementary Figure S4B), with the highest proportion in PE5 (73.0%), followed by CON (66.6%), and the lowest in PE40 (49.4%). *Bacteroidota* was the second most dominant phylum in PE10, PE20, and PE40 groups, but the proportion of *Actinobacteriota* was higher than *Bacteroidota* in CON and PE groups. *Olsenella*, *Prevotella*, *Lachnospiraceae_NK3A20_group*, *norank_f__norank_o__Clostridia_UCG-014*, and *Shuttleworthia* were the dominant ruminal bacterial genera at 75 d (Supplementary Figure S4C).

**Figure 4:**
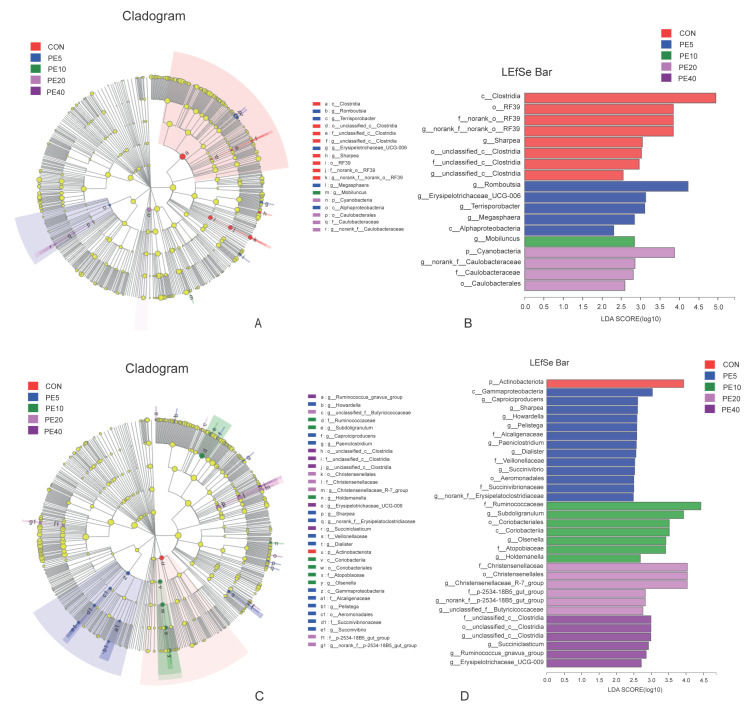
LEfSe analysis (LDA, threshold of 2) of fecal bacterial taxa that were significantly enriched among the groups of calves supplemented with different levels of Amla (*Phyllanthus emblica*) whole-fruit powder at (**A**,**B**) 28 days of age and (**C**,**D**) 75 days of age. CON, PE5, PE10, PE20, and PE40 indicate control (0), 5, 10, 20, and 40 g/d/head supplementation of Amla fruit powder, respectively.

LEfSe multilevel species difference discriminant analysis was employed to detect significantly different fecal bacterial taxa at the phylum to genus level among the treatment groups using the online Majorbio Cloud Platform (www.majorbio.com, accessed on 11 April 2022). First, the non-parametric factorial Kruskal–Wallis (KW) rank sum test was used to detect taxa with significant differences. Then, the effect size of each significantly abundant taxon was estimated using linear discriminant analysis (LDA). The LEfSe cladogram and linear discriminant analysis (LDA, threshold of 2) histogram for fecal samples are shown in Figure 4. No significant difference in taxa among treatments was detected at 2 d (Supplementary Figure S5A). However, at 28 d, the LEfSe analysis identified 18 significantly enriched taxa among the different treatments (Figure 4A,B). Treatments CON, PE5, PE10, and PE20 had significantly higher abundances of eight, five, one, and four taxa, respectively (Supplementary Table S1). Higher abundances of *c__Clostridia* in CON and *p__Cyanobacteria* in PE20 were detected, while the abundance of *c__Alphaproteobacteria* was higher in PE5. Similarly, 18 taxa that significantly differed among treatments were identified at 42 d; however, PE40 and PE10 were enriched with more taxa (eight and five taxa, respectively) than the other treatment groups (Supplementary Figure S5B). At 75 d, the abundances of 33 taxa were significantly different among the treatment groups (Figure 4C,D). The only taxon enriched in CON groups was *p__Actinobacteriota,* while PE5 had significantly higher abundance of 13 taxa, including *c__Gammaproteobacteria*. The abundances of seven taxa, including *f__Ruminococcaceae*, were higher in PE10, whereas PE20 and PE40 had higher abundances of six taxa each (Supplementary Table S2).

**Figure 5:**
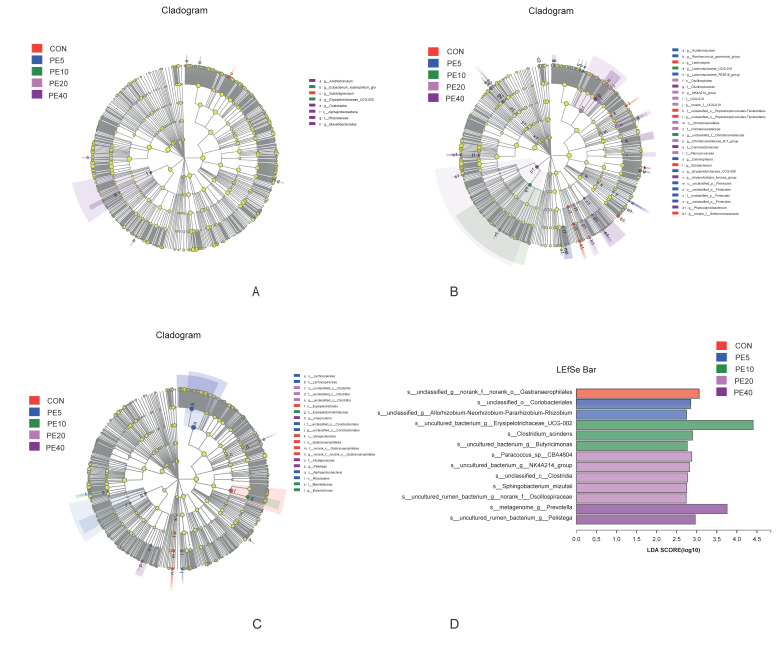
LEfSe analysis (LDA, threshold of 2) of rumen bacterial taxa that were significantly enriched among the groups of calves supplemented with different levels of Amla (*Phyllanthus emblica*) whole-fruit powder at (**A**) 14 days of age; (**B**) 42 days of age; (**C**,**D**) 75 days of age. CON, PE5, PE10, PE20, and PE40 indicate control (0), 5, 10, 20, and 40 g/d/head supplementation of Amla fruit powder, respectively.

The LEfSe analysis of rumen fluid samples revealed that only eight taxa were significantly abundant among the treatment groups, in which one, two, and five taxa were enriched in CON, PE10, and PE40, respectively, at 14 d (Figure 5A). At 42 d, 28 taxa appeared to be significantly abundant among the treatments, and 17 were in PE5 and PE20 (Figure 5B). At weaning (75 d), PE6 was enriched with six taxa, which included *o__Lachnospirales*, *f__Lachnospiraceae*, *c__Alphaproteobacteria*, and *o__Rhizobiales,* while CON was enriched with five taxa, including *o__Erysipelotrichales*, *c__Vampirivibrionia*, and *o__Gastranaerophilales* (Figure 5C). The analysis of rumen bacteria at 75 d, at the species level, detected 13 species of bacteria with significant differences among the treatments (Figure 5D).

## 4. Discussion

### 4.1. Effect of Amla Fruit Powder on Antioxidant Capacity

Maintaining the balance between antioxidants and free radicals is essential for the proper physiological functioning of the cell [31], whereas excess accumulation of free radicals causes oxidative stress, which can damage the structures and functions of cells [31,32]. Thus, antioxidants are important cell defense mechanisms through scavenging excessively produced free radicals [33]. Activities of antioxidant enzymes (GSH-Px, CAT, and SOD) are important indicators of the oxidative stress defense capacity in animals [34,35], and the concentration of MDA is among the commonly used oxidative stress biomarkers [35,36]. GSH-Px, CAT, and SOD are preventive antioxidants with the first two involved in the blocking of free radical production or the detoxification of free radicals during the early formation process [37,38], and the later inhibiting lipid peroxidation [37] or removing superoxide radicals by converting them to oxygen and hydrogen peroxide [H_2_O_2_] [34]. In the current study, PE supplementation at a low dose (PE5) improved the antioxidant capacity (activities of GSH-Px, CAT, and SOD) and reduced the oxidative stress (serum concentration of MDA) in preweaning dairy calves. This is in line with Saha and Verma [15], who reported that polyphenolic extract of Amla fruit protected against oxidative stress (decreased lead-acetate-induced peroxidation) and increased the activities of SOD, GSH, and CAT in rats due to its strong free radical scavenging activity. In agreement with the current study, Rajak et al. [39] revealed that chronic supplementation of fresh Amla fruit significantly increased the activities of SOD, CAT, and GSH-Px in rats, and protected the heart from ischemia–reperfusion-induced oxidative stress.

Shivananjappa and Joshi [40] reported that aqueous extracts of Amla significantly increased the activities of antioxidant enzymes (SOD and CAT) and reduced the production of free radicals, and thereby improved antioxidant defense in HepG2 cells. Similarly, in a study by Charoenteeraboon et al. [41], Amla fruit extract inhibited H_2_O_2_-induced oxidative stress in human myeloleukemic U937 cells. Usharani et al. [42] also reported that 250 mg and 500 mg *Emblica officinalis* capsules twice daily supplementation for humans improved the antioxidant status of type 2 diabetes patients (improved activities of glutathione and reduced MDA level). Fresh Amla fruit supplementation in dairy cows also improved the activity of SOD in blood [13]. The increased activity of SOD, along with increased activities of CAT and GSH-Px in the PE5 group, in the current study, is important as the balance of the antioxidants is essential for the body. For instance, SOD is an important antioxidant, but produces H_2_O_2_ as a metabolite, which is toxic unless scavenged by CAT and/or GSH-Px [39]. Most of the studies on the antioxidant effect of Amla fruit, other than the in vitro studies, used rats under alcohol-induced oxidative stress, and evaluated the effect of Amla fruit extracts on reducing oxidative stress and restoring antioxidant status. These studies reported that supplementation of Amla fruit extracts ameliorated alcohol-induced oxidative stress, toxicity, cell damage, and injury, and increased the activities of antioxidant enzymes GSH-Px, SOD, and CAT.

The improved antioxidant status and reduced oxidative stress in the current study could be due to the strong antioxidant capacity of Amla fruit. Several in vitro studies reported strong antioxidant capacities of extracts from Amla fruit. For example, [41,43] revealed that extracts of Amla fruit showed strong free radical scavenging and reactive oxygen species production inhibition capacity. The strong antioxidant capacity of Amla fruit could be due to its high contents of tannins and flavonoids. This is supported by Reddy et al. [44], who concluded that the effects of Amla fruit on antioxidant status are due to the combined effects of phytochemicals such as tannins, flavonoids, and vitamin C. Studies showed that tannins from different plant extracts showed strong antioxidant properties. Tannins can inhibit lipid peroxidation by chelating metal ions [45], scavenge free radicals [46], are involved in the metabolism of other antioxidants, and inhibit peroxidative enzymes [47].

In addition to PE5, PE10 also improved the antioxidant activities, but did not affect the oxidative stress biomarker MDA. However, higher doses (PE20 and PE40) caused increased oxidative stress compared to the CON, although activities of antioxidant enzymes were not affected, except CAT, which was lower in PE40. The increased oxidative stress in higher doses could be due to the toxic effect of Amla fruit at higher doses. In the current study, we used the whole fruit, including the seed. The seed has the highest tannin concentration of the fruit [48], and potentially more toxicity than the pulp [49]. Shivananjappa and Joshi [40] reported a cytotoxic effect of Amla extracts at a dose of >250 µg/mg, which reduced the viability of HepG2 cells. Tannins may have adverse effects at high doses. According to Middha et al. [50], tannin supplementation up to 400 mg/kg did not cause any toxicity in Wistar albino rats, although the median lethal dose was 1125 mg/kg BW.

### 4.2. Effect of Amla Fruit Powder on Immune and Anti-Inflammatory Responses

The overall results of the current study illustrate that PE supplementation at 5 g/d improved the serum immunoglobulins (IgA, IgG, and IgM) and anti-inflammatory cytokine (IL-10) levels in preweaning dairy calves without affecting the proinflammatory cytokine (TNF-α) level, compared to the control. However, serum concentrations of immunoglobulins and IL-10 decreased with increasing levels of PE, while the proinflammatory cytokine, TNF-α, increased with increasing PE levels. This is partially consistent with [43], who reported a biphasic effect of Amla fruit extract on the immunological variables. They reported that Amla fruit extract at 60 mg/kg modulated the pro- and anti-inflammatory cytokines in ulcerated mice by increasing the levels of IL-10 and decreasing TNF-α, while 40 mg/kg and 120 mg/kg had the reverse effects.

Activation of the immune response is an effective method of protection against various infectious agents [51]. IgM is the first immunoglobulin to participate in the immune response during microbial infection, followed by IgG and IgA, sequentially [52]. The higher immune response (higher serum concentrations of immunoglobulins) throughout the study in PE5 indicates that calves might have been under the continuous challenge of microbial infection, and that supplementation of PE5 triggered increased immune defense in response to these challenges. In agreement with this, tannins derived from Amla fruit boosted the immune response and protected broiler chickens against *Eimeria*-species-induced coccidial infection [53], which is also among the causes of diarrhea in calves [54]. Similarly, Tannin-containing feeds enhanced the immune response [55] and reduced preweaning mortality of lambs [56]. The higher IL-10 level in PE5 in the current study, compared to the control, may be attributed to the anti-inflammatory property of Amla fruit [18]. TNF-α level was not affected by PE5 compared to the CON, but it increased with increasing PE level, and was significantly higher at a higher dose (PE40). This, along with the low levels of immunoglobulins and IL-10 in PE40, indicates that PE supplementation at higher doses negatively affected the health of calves, and thereby increased levels of the proinflammatory cytokine TNF-α. This is in line with [43], who stated that the stimulation of proinflammatory cytokines, an important component of the mucosal defense, and decreasing anti-inflammatory cytokines (IL-10) are modes of mediation during exposure to challenging health conditions.

In general, the low dose (5 g/d) of Amla fruit powder supplementation improved the immune and anti-inflammatory capacity of preweaning dairy calves. This effect of Amla fruit on immune response may be due to the immunostimulatory effect of tannins [53]. The immunomodulatory effect of Amla fruit is also attributed to its role in protection against oxidative damage caused by free radicals and improving the levels of antioxidants and antioxidant enzymes [51]. Whereas the improved anti-inflammatory response could be due to pyrogallol, a bioactive compound produced during microbial degradation of hydrolyzable tannins in the rumen [57], which was found to be responsible for the anti-inflammatory effect of Amal fruit in a study by Nicolis et al. [58]. On the other hand, the immune response and anti-inflammatory capacity of calves decreased, and proinflammatory cytokine level increased, with increasing levels of PE. This negative health effect of a higher dose (PE40) could be due to the toxic effect of hydrolyzable tannins at higher levels. Most of the tannins contained in Amla fruit are hydrolyzable tannins [51], and accumulated hydrolyzable tannins can be potentially toxic to ruminants [57].

### 4.3. Effect of Amla Fruit Powder on Rumen and Fecal Bacterial Structure

Gut microbiota play a significant role in maintaining proper physiological hemostasis and regulating the development and maturation of the immune system at early age [59,60]. On the other hand, disturbance in the balance of rumen microbial community (dysbiosis) causes severe adverse effects, which include colonization of pathogenic bacteria in the gut [61] and reduction in the abundance of beneficial microbes [62], bacterial translocation across the gut barrier [63], and release of toxins and metabolites into the blood and tissue [60]. These adverse effects lead to an increased inflammatory response and related health problems [60,64]. In the current study, the 16S rRNA bacterial genome analysis of rumen fluid and fecal samples of preweaning dairy calves supplemented with PE revealed that no significant effect was observed for the bacterial diversity and richness, with the exception of the higher Simpson index of fecal samples detected in PE10 compared to the CON and PE20 at 28 days of age. However, significant differences in the abundance of the ruminal and fecal bacterial community among treatment groups were detected, suggesting that the effects of PE supplementation were primarily on the dynamics of gut bacterial community.

The higher proportion of *Firmicutes* in PE5 compared to the other groups at early age in the current study might have helped the development of the immune system in the PE5 group, as *Firmicutes* plays an important role in the regulation of host immunity [65]. Studies also revealed that *Firmicutes* and *Bacteroidetes* improve the production of IL-10, and thereby contribute to the host defense system. The activation of IL-10 production is one of the roles of the gut microbiota in the host’s defense against pathogens [66]. Many species of bacteria in the *Firmicutes* phylum are also known for their role in breaking down fiber and resistant starch in the gut [67]. The presence of higher proportions of *Proteobacteria*, which are potentially pathogenic [68], in CON and PE20 might have contributed to the reduced health status of calves in these groups compared to the PE5 treatment group. In line with this, Frank et al. [69] reported that increases in *Proteobacteria* and *Actinobacteria*, and decreases in *Firmicutes* and *Bacteroidetes*, are associated with inflammatory diseases.

The higher fecal concentration of *Prevotella* in the PE5 group at 75 d may also be associated with the better health status of calves in PE5 compared to the other treatment groups. In support of this, in situ and in vitro studies by Schogor [70] suggested that *Prevotella* spp. may contribute a main role in the conversion of lignans into health-beneficial antioxidants. According to Khafipour [71], some species of *Prevotella* may prevent subacute ruminal acidosis and the disruption of the bovine digestion process by preventing the colonization of acid-producing bacteria. These all might have contributed to the better health status of the calves supplemented with PE5. The higher abundance of *Prevotella* in the rumen is also associated with reduced methane emissions [72], which suggests that Amla fruit powder could be an environmentally friendly solution to improve ruminant health status, though this needs further investigation.

Studies showed that the gut *Bacteroidetes* to *Firmicutes* ratio may indicate the serum immunoglobulin levels. According to Shen et al. [73], the gut *Bacteroidetes* to *Firmicutes* ratio in humans was found to be positively correlated with serum concentrations of IgG and IgM. In the current study, the fecal *Bacteroidetes* to *Firmicutes* ratio at weaning (75 d) was higher in PE5, followed by CON, and was lower in PE20. This could be partially associated with the higher serum immunoglobulin concentrations detected in PE5 in the current study.

## 5. Conclusions

Supplementation of Amla (*Phyllanthus emblica*) fruit powder at 5 g/d protected preweaning dairy calves against oxidative stress and enhanced the antioxidant capacity, and immune and anti-inflammatory responses. However, high doses (20 and 40 g/d) of PE supplementation decreased the serum concentration of the antioxidant enzymes, immune response indices, and IL-10 level, along with increased MDA and TNF-α, indicating an immunosuppressive and proinflammatory effect at higher doses. No significant variation was detected in the fecal and ruminal bacterial diversity and richness of calves supplemented with different levels of PE. However, significant differences in the fecal and ruminal bacterial community abundances, such as higher proportion of *Firmicutes* at early age and higher *Bacteroidetes* to *Firmicutes* ratio at weaning (75 d), were detected in the PE5 group. The overall study results suggest that supplementing 5 g/d Amla (*Phyllanthus emblica*) fruit powder may improve the antioxidant capacity, and immune and anti-inflammatory responses, while maintaining normal gut bacterial hemostasis. Further investigation into the effect of Amla fruit on the whole gut microbiota structure and mode of action is recommended.

## Figures and Tables

**Table 1:** Chemical composition of experimental diets used in the study.

Items	Calf Starter ^1^	Whole Milk	Milk Replacer ^1^	Amla (*Phyllanthus emblica*) ^1^
Ingredients (% of DM):		-	-	-
Steam-flaked corn	40.50	-	-	-
Corn gluten	2.54	-	-	-
Soybean meal	20.51	-	-	-
Extruded soybean	6.10	-	-	-
Wheat bran	5.86	-	-	-
Wheat shorts	7.12	-	-	-
Canola meal	11.78	-	-	-
Cane molasses	1.66	-	-	-
Calf starter premix ^2^	3.93	-	-	-
Chemical composition:		-	-	-
DM (%)	92.90	-	95.79	93.53
Ash (%)	6.49	-	7.22	3.54
CP (%)	23.88	-	22.45	6.64
EE (%)	3.19	-	8.99	1.22
NDF (%)	16.89	-	-	36.25
ADF (%)	6.93	-	-	28.15
Density (g/L)	-	1030.78	-	-
Milk protein (%)	-	3.54	-	-
Milk fat (%)	-	3.76	-	-
Total solid (%)	-	12.90	-	-
Lactose (%)	-	4.70	-	-

^1^ In % of DM. ^2^ Contained per kg of starter premix: vitamin A, 13,048 IU; vitamin D, 3269 IU; vitamin E, 261.005 IU; Fe, 116.822 mg; Cu, 19.627 mg; Mn, 48.602 mg; Zn, 74.616 mg; Se, 0.785 mg; I, 1.355 mg; and Co, 0.938 mg.

**Table 2:** Serum immunoglobulin and cytokines concentrations of calves supplemented with Amla (*Phyllanthus emblica*) whole-fruit powder.

Items ^1^	Treatments ^2^					SEM	*p*-Value ^3^		
	CON	PE5	PE10	PE20	PE40		Trt	D	Trt × D
IgA (µg/mL)									
Overall	651.1 ^b^	723.1 ^a^	710.3 ^a^	616.8 ^bc^	601.7 ^c^	11.63	<0.001	0.099	0.294
D28	641.8 ^ab^	687.3 ^a^	688.2 ^a^	615.6 ^b^	610.9 ^b^	15.92	0.003		
D75	660.4 ^bc^	758.9 ^a^	732.5 ^ab^	617.9 ^c^	592.4 ^c^	22.22	<0.001		
IgG (µg/mL)									
Overall	6221.1 ^b^	6977.5 ^a^	6830.2 ^a^	5743.6 ^c^	5729.8 ^c^	112.14	<0.001	0.263	0.379
D28	6142.3 ^bc^	6757.0 ^a^	6715.9 ^ab^	5915.5 ^c^	5698.1 ^c^	143.93	<0.001		
D75	6316.6 ^bc^	7197.9 ^a^	6944.4 ^ab^	5605.2 ^c^	5761.6 ^bc^	198.49	<0.001		
IgM (µg/mL)									
Overall	200.7 ^b^	227.9 ^a^	222.7 ^a^	192.6 ^b^	188.0 ^b^	4.29	<0.001	0.017	0.191
D28	190.4 ^bc^	216.8 ^a^	215.6 ^ab^	193.9 ^abc^	188.6 ^c^	6.14	0.004		
D75	211.1 ^bc^	138.9 ^a^	229.7 ^ab^	191.1 ^c^	187.5 ^c^	6.45	<0.001		
TNF-α (µg/mL)									
Overall	0.361 ^bc^	0.252 ^c^	0.280 ^c^	0.446 ^ab^	0.480 ^a^	0.03	<0.001	0.123	0.974
D28	0.333 ^ab^	0.236 ^b^	0.254 ^b^	0.427 ^ab^	0.475 ^a^	0.05	0.008		
D75	0.389 ^ab^	0.267 ^b^	0.306 ^b^	0.468 ^a^	0.485 ^a^	0.03	<0.001		
IL-10 (pg/mL)									
Overall	38.80 ^bc^	52.21 ^a^	46.78 ^ab^	39.42 ^bc^	35.57 ^c^	1.93	<0.001	0.486	0.584
D28	34.71 ^b^	51.45 ^a^	47.99 ^ab^	38.68 ^ab^	36.14 ^ab^	2.70	0.011		
D75	42.90 ^bc^	52.97 ^a^	45.57 ^ab^	40.15 ^bc^	34.99 ^c^	2.32	<0.001		

^1^ IgA = immunoglobulin A; IgG = immunoglobulin G; IgM = immunoglobulin M; TNF-α = tumor necrosis factor α; IL-10 = interleukin 10. ^2^ CON, PE5, PE10, PE20, and PE40 indicate control (0), 5, 10, 20, and 40 g/d/head supplementation of Amla fruit powder, respectively. ^3^ Trt = treatment; D = day; Trt × D = treatment–day interaction. ^a–c^ Means with different letters within a row are significantly different (*p* < 0.05).

## Data Availability

Data contained within the article, Supplementary Materials, and the raw sequencing reads can be accessed from the NCBI Sequence Read Archive (SRA) database (BioProject ID: PRJNA886656).

## Supplementary Materials

The following supporting information can be downloaded at: https://www.mdpi.com/article/10.3390/biology11121753/s1. Table S1: Fecal bacterial taxa that significantly differed among treatments by LEfSe at 28 days of age. Table S2: Fecal bacterial taxa that significantly differed among treatments by LEfSe at 75 days of age. Figure S1: Fecal bacterial diversity at 2 and 42 days of age. Figure S2: Rumen bacterial diversity at 14, 42, and 75 days of age. Figure S3: Relative abundance of rumen bacterial community. Figure S4: LEfSe analysis (LDA, threshold of 2) of fecal bacterial taxa. Figure S5: LEfSe analysis (LDA, threshold of 2) of fecal bacterial taxa in calves supplemented with different levels of Amla (*Phyllanthus emblica*) whole fruit powder at: (A) 3 days of age; (B) 42 days of age; CON, PE5, PE10, PE20, and PE40 indicate control (0), 5, 10, 20, and 40 g/d/head supplementation of Amla fruit powder, respectively.
