# Supplementary figures and images for: Phyllanthus emblica (Amla) Fruit Powder as a Supplement to Improve Preweaning Dairy Calves’ Health: Effect on Antioxidant Capacity, Immune Response, and Gut Bacterial Diversity
Source: Biology (Basel). 2022 Dec 1;11(12):1753. doi: 10.3390/biology11121753 (PMC9774823; doi:10.3390/biology11121753)

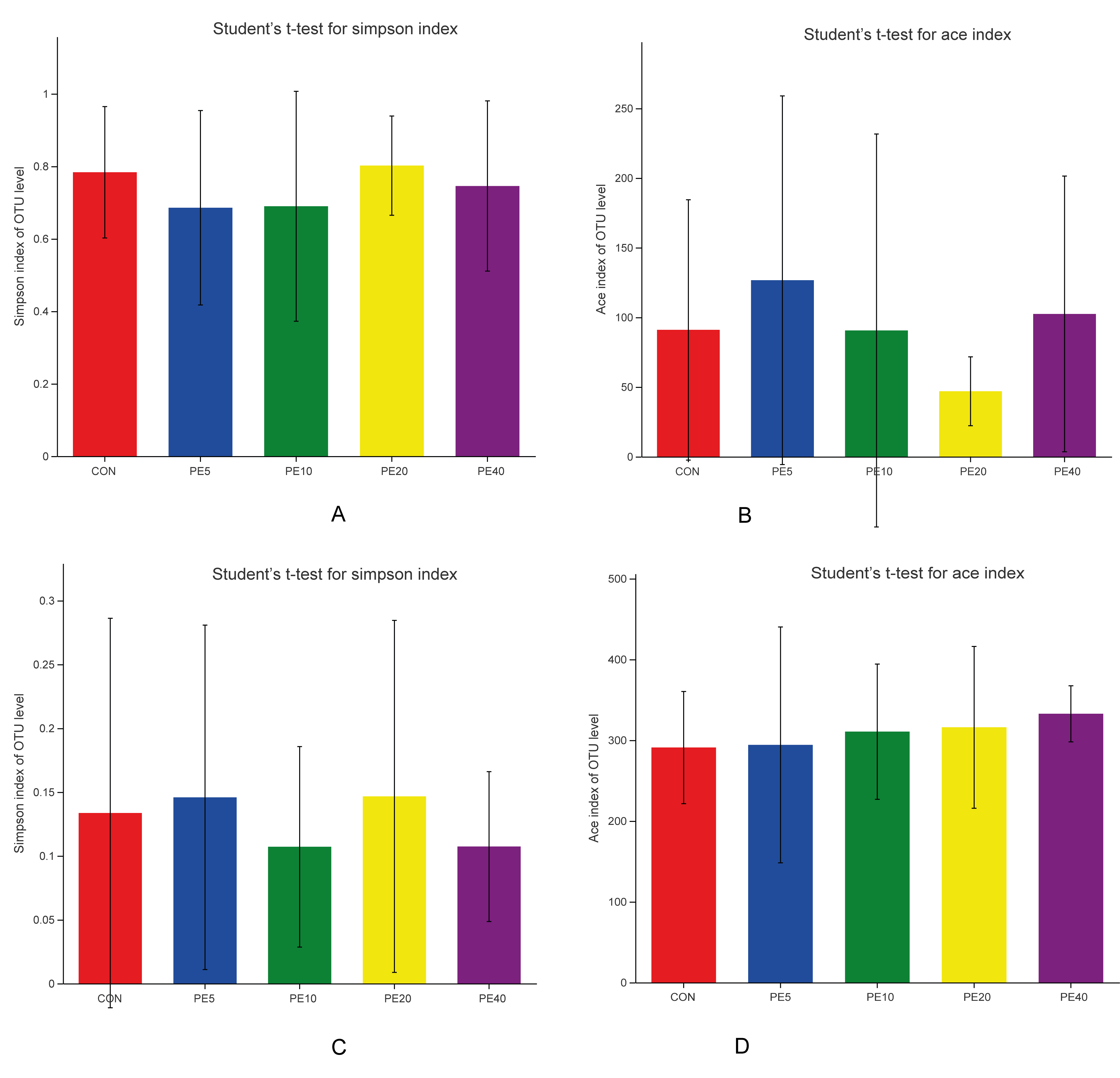

Supplement: Supplementary file 1 [file biology-11-01753-s001.zip › Figure S1.tif]

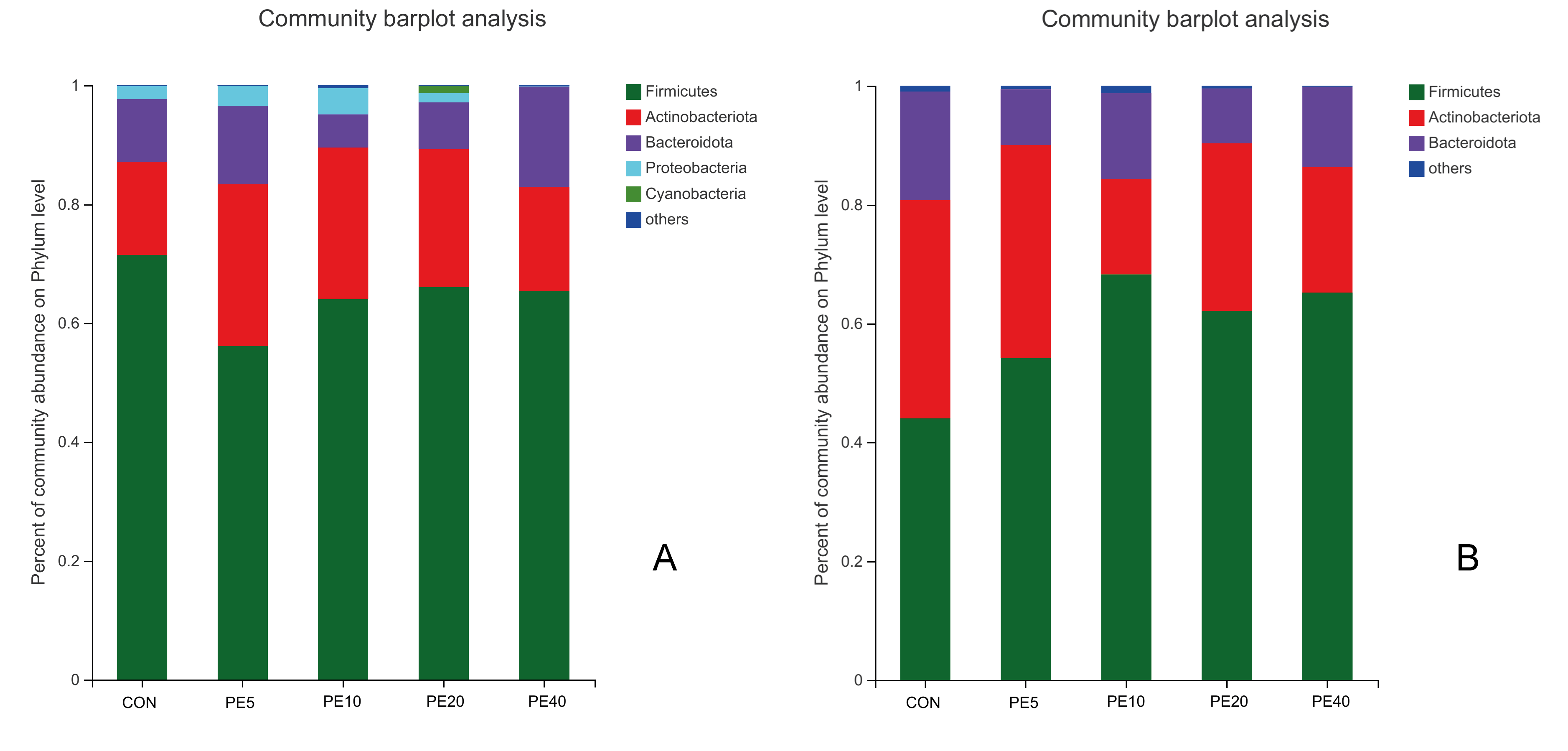

Supplement: Supplementary file 1 [file biology-11-01753-s001.zip › Figure S3.tif]

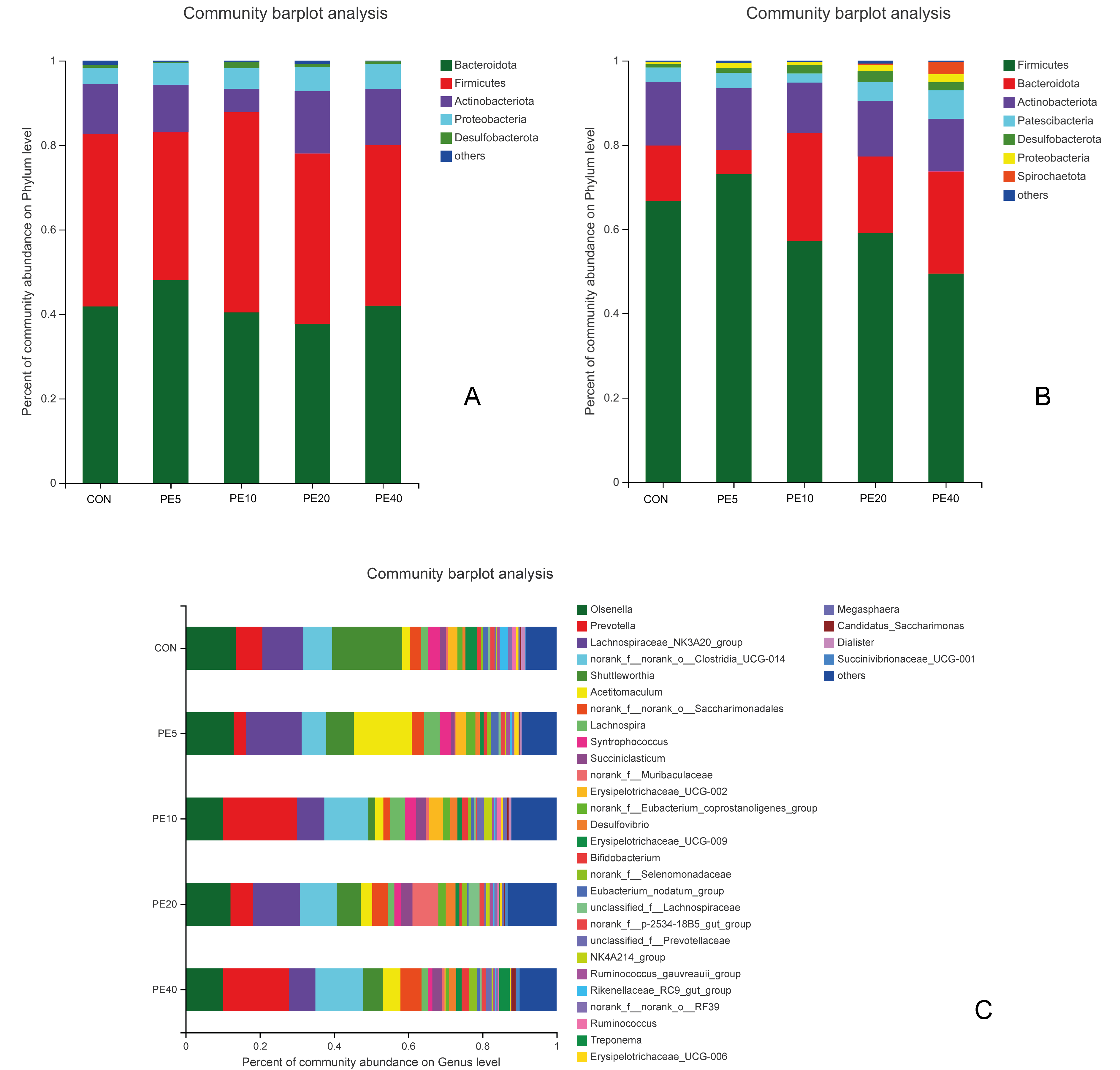

Supplement: Supplementary file 1 [file biology-11-01753-s001.zip › Figure S4.tif]

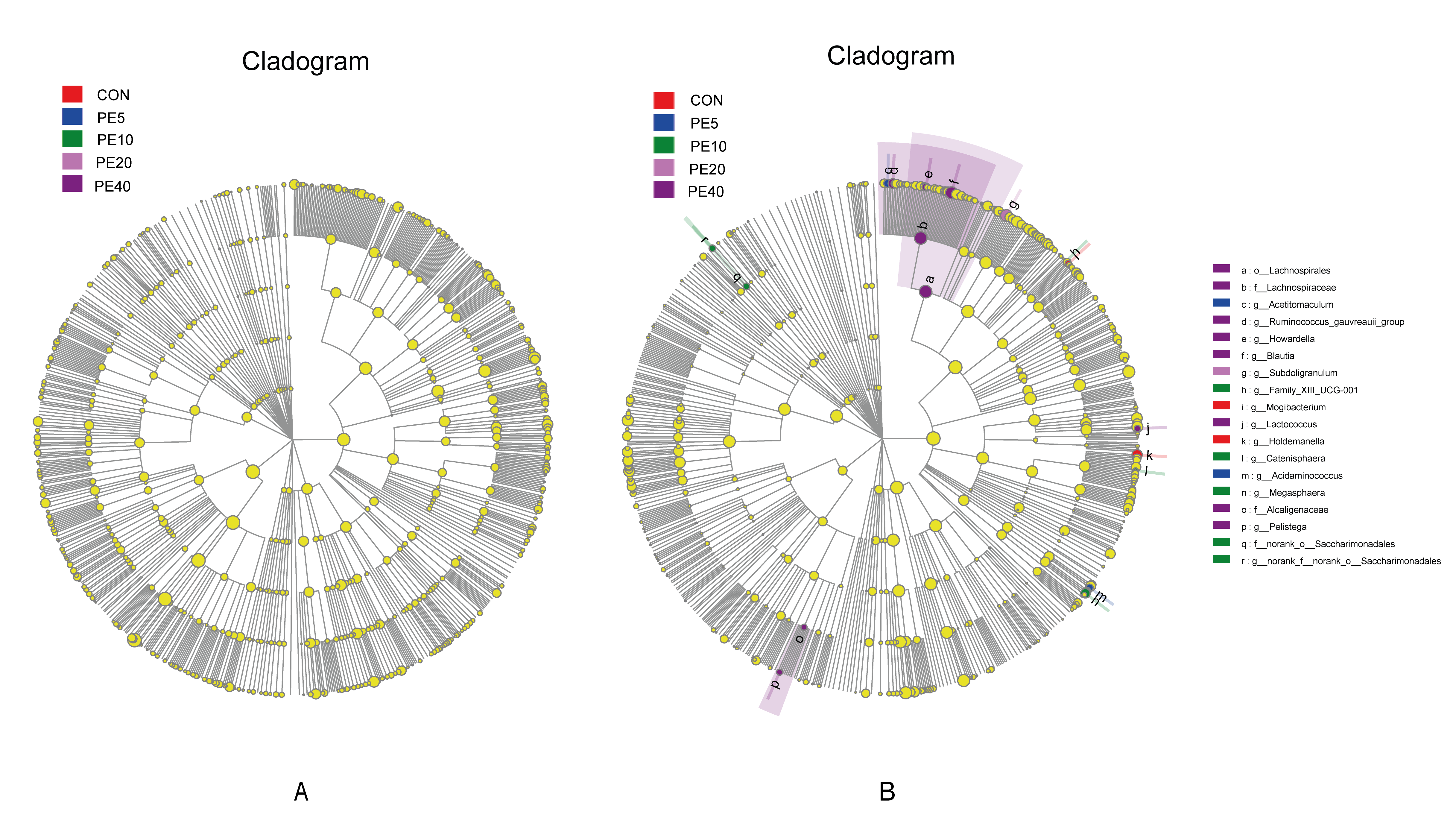

Supplement: Supplementary file 1 [file biology-11-01753-s001.zip › Figure S5.tif]
